# Predictive Modeling of Critical Temperatures in Superconducting Materials

**DOI:** 10.3390/molecules26010008

**Published:** 2020-12-22

**Authors:** Natalia Sizochenko, Markus Hofmann

**Affiliations:** 1 Department of Informatics, Blanchardstown Campus, Technological University Dublin, 15 YV78 Dublin, Ireland; markus.hofmann@tudublin.ie; 2 Department of Informatics, Postdoctoral Institute for Computational Studies, Enfield, NH 03748, USA

**Keywords:** critical temperature, thermal conductivity, predictive modeling, QSPR, machine learning

## Abstract

In this study, we have investigated quantitative relationships between critical temperatures of superconductive inorganic materials and the basic physicochemical attributes of these materials (also called quantitative structure-property relationships). We demonstrated that one of the most recent studies (titled "A data-driven statistical model for predicting the critical temperature of a superconductor” and published in Computational Materials Science by K. Hamidieh in 2018) reports on models that were based on the dataset that contains 27% of duplicate entries. We aimed to deliver stable models for a properly cleaned dataset using the same modeling techniques (multiple linear regression, MLR, and gradient boosting decision trees, XGBoost). The predictive ability of our best XGBoost model (R2 = 0.924, RMSE = 9.336 using 10-fold cross-validation) is comparable to the XGBoost model by the author of the initial dataset (R2 = 0.920 and RMSE = 9.5 K in ten-fold cross-validation). At the same time, our best model is based on less sophisticated parameters, which allows one to make more accurate interpretations while maintaining a generalizable model. In particular, we found that the highest relative influence is attributed to variables that represent the thermal conductivity of materials. In addition to MLR and XGBoost, we explored the potential of other machine learning techniques (NN, neural networks and RF, random forests).

## 1. Introduction

Superconducting materials are capable to conduct electric current with zero resistance at or below a certain critical temperature TC [1]. Since the very first discovery of superconductivity in mercury, thousands of elements and alloys were found to express superconducting properties [2]. Several theories analyze how superconductivity got established in materials. For example, the commonly accepted Bardeen–Cooper–Schrieffer theory of superconductivity attributes the manifestation of superconductivity in a given material to the formation of resonant states of electron pairs [3,4,5]. It could be discussed in the context of the formation of ions that move through the crystalline lattice of the superconductor [6].

The phenomenon of superconductivity is widely applied in the industry: for example, superconductors are used to create powerful electromagnets, electrical systems, etc. Engineers generally follow empirical rules to create and test new superconducting materials. However, such an approach is not systematic and therefore could be time-consuming and expensive. A potential solution is to apply computational techniques, such as multiphysics simulations to study superconducting effects in materials [7]. At the same time, sophisticated physics-based modeling algorithms require significant computing resources and are not suitable for fast predictions.

In recent years, with the emergence of structured databases for materials, scholars directed their efforts toward the development of predictive models for physicochemical properties and biological activities [8]. An application of methods of machine learning could help to facilitate the discovery of novel materials based on data for known materials [9]. In the field of superconducting materials, the creation of fast predictive tools will reduce the final cost of production of superconductors with the desired critical temperatures. In addition to that, predictive modeling in a materials science context could aid experimental teams in their search for superconductors with desired properties. Moreover, the use of data-driven predictive modeling could help to reduce the number of lengthy and expensive experiments or complex physics-based computational simulations [10,11,12]. Such machine learning-based models in chemistry are generally called the Quantitative Structure-Property Relationship (QSPR) models and they usually serve as an efficient tool for fast screenings and properties prediction [13]. Popular algorithms used in QSPR moldings these days include multiple linear regression (MLR), principal component analysis (PCA), projections to latent structures (PLS), random forests (RF), decision trees (DT), artificial neural networks (ANN), and many others [14,15,16].

The most recent studies suggest that the chemical information could be successfully integrated with techniques of machine learning [8,10,11,12,17,18,19]. A series of predictive models that explore quantitative relationships between critical temperature and physicochemical properties of materials have been reported in the literature [1,6,20,21]. One of the pioneering works directly attributes critical temperatures of 60 high-temperature superconductors to valence-electron numbers, orbital radii, and electronegativity [21]. Later, PCA and PLA were applied to predict TC for 1212 superconductive copper oxides [20]. Most recently, predictive and classification models were generated for more than 10,000 known superconductors using the RF, MLR, and gradient boosting techniques [1,6].

The goal of this article is to deliver models that accurately predict the critical temperatures for inorganic superconducting materials. We used the dataset that contains information about 21,263 inorganic superconductors, as reported by K. Hamidieh [1]. We also aimed to compare our models to existing models developed for the same dataset, and to provide insights into the most influential physicochemical attributes. Finally, we discussed developed models in the context of potential applications in materials science.

## 2. Results and Discussion

### 2.1. Data Pre-Processing

**Figure 1:**
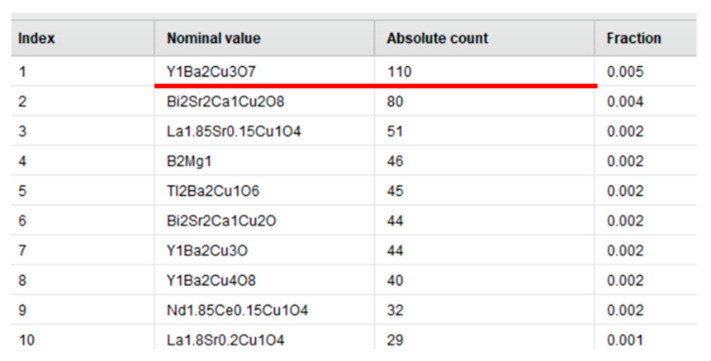
Example of duplicate measurements for the same material: nominal value—a type of material, absolute count—number of duplicate measurements, fraction—the number of duplicates for every material in relation to the total number of entries in the dataset.

At first sight, the initial dataset did not contain any duplicates. However, after careful examination, we found that the data contained a lot of similar TC values for the same material. Examples of duplicate measurements extracted are presented in Figure 1.

**Figure 2:**
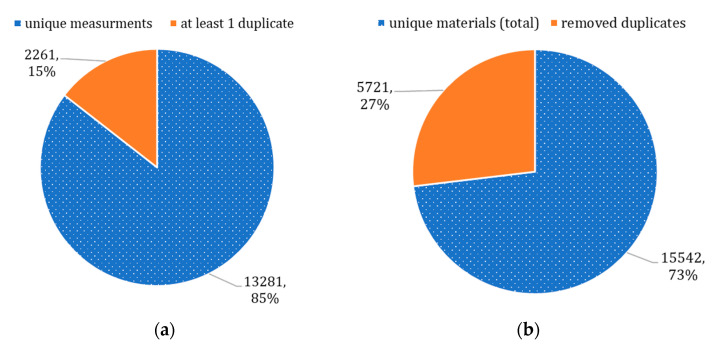
Identified duplicates: (**a**) unique and duplicate measurements, (**b**) updated dataset.

Overall, we found that 85% of materials had a single T_C_ measurement reported (Figure 2a), and the remaining materials had at least 1 duplicate entry reported (e.g., 1331 materials had two values of T_C_ reported). A total of 7982 duplicates were identified for 2261 materials in total, and only 15,542 materials were truly unique (Figure 2b). This issue occurred because the dataset contained a compilation of T_C_ measurements reported by different research teams. The variation of measurements for the same material could either happen because measurements were conducted for different types of crystal structures or simply because of an instrumental error. In conclusion, specific domain knowledge is likely required for the data collection and preparation in this area of knowledge; otherwise, data science specialists might not be able to identify quality issues.

We have removed duplicates as discussed in the *Materials and Methods* section. The dataset with removed duplicates is further referred to as a “cleaned dataset” or simply a “dataset”. An overview of the cleaned dataset is presented in Supplementary Materials (Table S2). The cleaned dataset did not contain constants or near-constant attributes, and the variability of each attribute was adequate.

### 2.2. Model Development

All the models discussed in this section could be downloaded from the Supplementary Materials file (Models S4).

**Figure 3:**
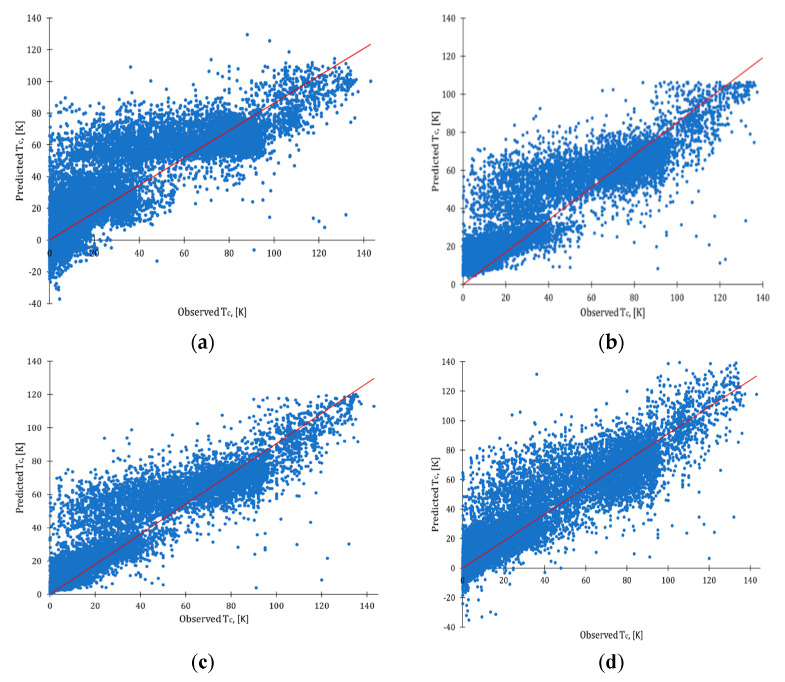
Observed vs. predicted plots: (**a**) baseline MLR model; (**b**) baseline XGBoost model; (**c**) baseline RF model; (**d**) baseline NN model; red line represents ideal fit.

First, baseline predictive models using the cleaned dataset were developed, applying default settings of nodes. All models discussed here were validated using a 10-fold cross-validation technique (see details in *Materials and Methods* section). Statistical characteristics and observed vs. predictive plots for baseline models are presented in Figure 3. As could be seen, baseline models for MLR and NN reported multiple cases of negative values of T_C_ (such values of temperatures are physically impossible). Hamidieh [1] had a similar observation for their MLR and XGBoost models. XGBoost and RF baseline models predicted values for T_C_ in the positive range of temperatures (from 0 K to 140 K). At the same time, however, XGBoost and RF baseline models overpredicted values of Tc in a zone of low-temperature superconductors.

Next, we decreased the number of attributes as the relative importance of key attributes that could be influenced by co-dependent attributes in the dataset. To reduce the influence of unwanted co-dependencies, we used such preselection techniques, as weight by correlation, weight by relief, and weight by PCA. For the PCA, we found that the cumulative proportion of variance became optimal for 3 components (refer to Supplementary Materials, Figure S3). Finally, we identified and removed 685 outliers and repeated the modeling. Statistical characteristics of developed models are presented in Table 1, Table 2, Table 3 and Table 4.

The interpretation of Table 1, Table 2, Table 3 and Table 4 reveals that R^2^ values for developed models were in the range of 0.603–0.868. The preliminary removal of correlated attributes led to a decrease in quality. Similarly, the prioritization of attributes using weighting techniques did not improve the quality of models. A potential reason for that is an ineffective selection of attributes or dissatisfactory selection of modeling parameters. At the same time, the models that used the top-20 attributes selected by weighing by correlation filter were of higher quality compared to the models generated using weighting by PCA and weighting by relief filters. Once outliers were removed, the quality of some models improved. In fact, the best models for each algorithm were obtained for a dataset with removed outliers (marked in bold in Table 1, Table 2, Table 3 and Table 4).

Next, we used aggregated parameters to develop predictive models (Table 5). The predictive ability of models that contained aggregated attributes was only lower compared to the models discussed earlier. As can be seen, the statistical quality of the majority of MLR models was below acceptable limits (R^2^ > 0.6), while the quality of RF models was closer to models developed for the cleaned dataset. One of the reasons for decreased quality is the decline of the natural complexity of the data after aggregation. In other words, aggregated parameters are not fully capable to capture the hidden patterns of explored data. We then merged the aggregated attributes with the initial set of attributes, and the quality of models has improved and reached a level similar to the quality of models reported in Table 1, Table 2, Table 3 and Table 4. Unfortunately, this rather means that aggregated attributes did not add much value to the predictive ability.

### 2.3. Optimization of the Best Models

After careful examination of the discussed models, we can conclude that the quality of MLR models will not likely improve. MLR generates linear equations, and with the reduced number of attributes, the predictive ability will only decline. Our best MLR model is similar to Hamidieh’s model [1] in terms of statistical quality: R^2^ = 0.735 and RMSE = 17.409 K (our model) versus R^2^ = 0.74 and RMSE = 17.6 K (Hamidieh’s model).

At the same time, XGBoost, RF, and NN methods could potentially be improved with parameter tuning. For this article, we decided to focus on the XGBoost algorithm. There were two reasons for that. First of all, we aimed to use the least unambiguous algorithm for further mechanistic interpretation [22]. Secondly, as we aimed to outperform the XGBoost model developed by Hamidieh [1] using the smaller number of attributes and less sophisticated tuning parameters. The model reported in the literature and optimized models for both cleaned and uncleaned dataset are presented in Table 6.

Hamidieh’s XGBoost model was developed on data with duplicates; it included all 81 attributes and was tuned using 374 trees with the maximum depth of trees equal to 16 [1]. Table 6 shows that our models (even for a dataset with duplicates) generally outperformed the model by Hamidieh [1]. Specifically, our best model had lower RMSE and AE by 6.03% and 9.12%, respectively (Table 6, in bold). We suggest that there is still room for improvement, as optimization XGBoost models were built using a relatively small number of trees and the predictive quality could potentially be improved.

The optimal tuning parameters for XGBoost models were as follows: 20 attributes mapped to 50 trees of 16 maximal. We observed that, for optimized models, the quality has improved when highly correlated attributes have been preliminarily removed. The situation was the opposite in non-optimized models (Table 1, Table 2, Table 3 and Table 4). Next, the decrease of quality was insignificant when we switched from the full set of attributes to top-20 attributes. Hence, we can conclude that the reduced number of attributes is still capable to preserve and represent hidden patterns in data. Removal of outliers has slightly increased the quality of models.

**Figure 4:**
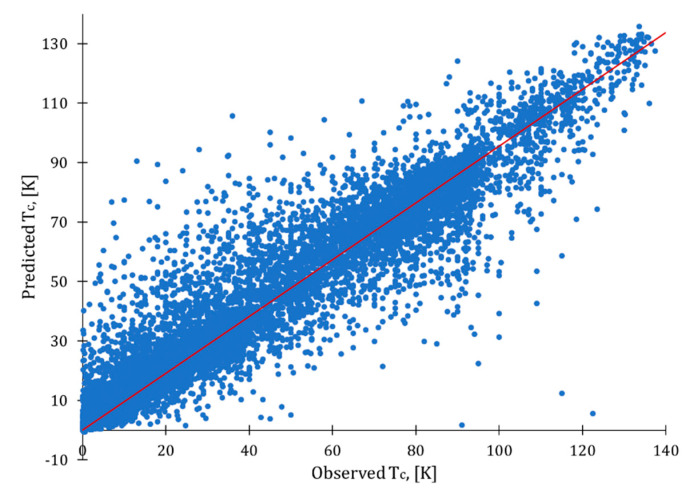
Observed vs. predicted plot for optimized XGBoost model.

For the data with no duplicates, the best optimized model was developed using all attributes with removed outliers (Table 6, in bold). Among the models with a reduced number of attributes, the best results were obtained with weight by relief for data with removed correlations, absence of outliers, weight by relief. It is clear from the observed-predicted plot (Figure 4) that there is still room for improvement, as some values were not predicted adequately (see dots located far from the ideal fit line in red). However, this model could still serve for a preliminary selection of superconducting materials.

### 2.4. Interpretation of Optimized Model and Potential Real-World Applications

**Figure 5:**
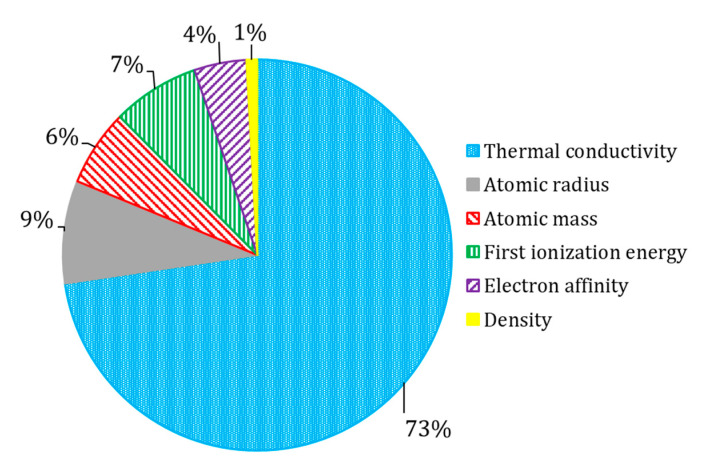
The relative influence of physicochemical parameters of selected XGBoost model.

The list of top-20 attributes and their importance are presented in Table 7. In order to generalize the interpretation, selected attributes were combined into groups (Figure 5). We found that the most influential attributes were related to thermal conductivity. This observation is in agreement with the observation by the author of the original dataset [1]. This is quite an expected outcome, as both superconductivity and thermal conductivity are driven by lattice phonons and electrons transitions [3]. The contribution of the first ionization energy could be explained with the Bardeen–Cooper–Schrieffer theory of superconductivity [3,4]. At the same time, ionic properties (related to the first ionization energy, and electron affinity) could likely reflect the capability of superconductors to form ions, that became involved in the movement through the crystalline lattice [6]. This interpretation also aligns well with Bardeen–Cooper–Schrieffer theory of superconductivity [3,4]. Attributes related to atomic properties and density represent intensive properties; their properties do not change when the amount of material in the system changes. Considering the nature of these attributes, they do not directly represent a physical process in superconductors, but rather reflect unique fingerprint-like features of chemical compounds [23].

Equipped with the knowledge about the physicochemical features that seem to be responsible for the T_c_ (Figure 5), the researchers working in the area of superconducting materials could prioritize materials with desired critical temperatures. This is especially important for the development of hybrid ferromagnetic/superconductor materials for spintronic applications [24,25].

The Supplementary Materials section contains the RapidMiner archive (Model S4 file), so that those readers interested in predicting T_C_ of the compound could benefit from using our models. It worth noting that our models are not without limitations: since the analyzed dataset did not contain doped and other hybrid materials, the prediction of T_C_ values might not be accurate enough. However, we encourage our readers to challenge our models with such predictions.

## 3. Materials and Methods

### 3.1. Dataset

The studied dataset was taken from the original research article by K. Hamidieh [1], deposited in the University of California Irvine data repository [26]. The original data were retrieved from the online database for superconducting materials called SuperCon, which is a comprehensive compilation of hundreds of research reports [27]. The dataset contains information on 82 physicochemical features (including critical temperature) for 21,263 superconductors [26]. All attributes are numeric and represent simplified physicochemical properties, calculated based on the chemical formula, such as a number of unique elements in a material, and sets of attributes that represent atomic mass, first ionization energy, atomic radius, density, electron affinity, fusion heat, thermal conductivity, and valence. In this dataset, the values of the first ionization energy were retrieved from http://www.ptable.com. The remaining attributes were generated with function ElementData in from Mathematica Version 11.1 by Wolfram and Research [28]. For more details on calculated attributes please refer to the original article [1]. A basic overview of the initial dataset is presented in Supplementary Materials (Table S1).

### 3.2. Duplicates Removal

The duplicates were first isolated from the dataset. For each material that contained a series of duplicate values of T_C_, we have analyzed the distribution of T_C_ measurements and removed data points with a standard deviation >5 K (for high-temperature superconductors with T_C_ > 10) or >2 K (for low-temperature superconductors with T_C_ < 10). For the remaining measurements, we have calculated the mean and then used that as a new T_C_ value. The procedure of duplicates removal was performed with the use of Python 3.5 [29].

### 3.3. Attribute Selection

Data were prepared for modeling using various attribute selection techniques. First, we have identified intercorrelations between attributes. We suggested that the removal of highly correlated attributes could help reducing redundancy. Once the desired level of intercorrelations (measured by the Pearson correlation coefficient) was set to <0.95, the number of attributes decreased from 81 to 60.

To further reduce the number of attributes for the modeling, we have pre-selected attributes using weighting by relief, by PCA, and by correlation. All preselection techniques were set to select the top-20 attributes to deliver a predictive model. Filtering by correlation is one of the most popular techniques [16]. Weighting by relief was selected, as this technique is both one of the most easily interpretable and successful algorithms to assess the quality of feature selection. Finally, PCA was selected as the author of the initial version of the dataset tried to apply this technique to reduce the number of attributes [1]. However, the author of the original article has abandoned this approach, explaining that the application of PCA was not beneficial.

We also attempted to reduce the number of attributes by introducing new aggregated attributes that represent a certain category of physical properties (e.g., atomic mass-related aggregation, thermal conductivity-related aggregation, etc.). As values of attributes are in different scales, we first normalized the dataset and then applied an average function to create aggregated attributes. The performance of models was tested using both initial attributes, aggregated attributes, and their mix.

Finally, we have analyzed if the dataset contained any outliers using the local outlier factor approach with a cut-off set at 3. These outliers were potentially a subject of removal.

### 3.4. QSPR Modeling

To develop the best QSPR model, we followed recommendations by OECD, considering the following five criteria: (i) a defined endpoint; (ii) an unambiguous algorithm; (iii) a defined domain of applicability; (iv) appropriate measures of goodness-of-fit, robustness, and predictive ability and (v) a mechanistic interpretation [22].

Similarly to the author of the initial dataset [1], we applied MLR and gradient-boosted decision trees (XGBoost) to develop predictive models. MLR expresses the dependency between attributes and target activity/property in a form of a simple mathematical function [30]. XGBoost delivers a model in a forming consensus of predictive decision trees ranked by the loss function [31].

In addition to the mentioned algorithms, we evaluated the performance of two other techniques: random forest (RF) and neural networks (NN). RF generates a collection of decision trees in the same way as XGBoost, however, the RF algorithm does not discriminate between trees, so all the trees contribute equally [32]. Finally, NN transforms input data into the hidden layers using different fitting techniques [30].

All models were validated using a 10-fold cross-validation technique: the dataset was split iteratively (10 times) into training and test subsets in a 9:1 ratio and the average performance of 10 resultant models was reported. Results were evaluated using squared correlation (R^2^), root mean squared error (RMSE), and absolute error (AE):

(1) $$R^{2}=1-\frac{\sum_{i=1}^{N}{\left({\hat{y}}_{i}-y_{i}\right)}^{2}}{\sum_{i=1}^{N}{\left(y_{i}-\tilde{y_{i}}\right)}^{2}}$$

(2) $$RMSE=\sqrt{\frac{\sum_{i=1}^{N}{\left({\hat{y}}_{i}-y_{i}\right)}^{2}}{N}}$$

(3) $$AE={\left({\hat{y}}_{i}-y_{i}\right)}^{2}$$

where N is the size of the test set, and$\;{\hat{y}}_{i}$, $y_{i}$, and $\tilde{y_{i}}$ are the correspondingly predicted, observed, and mean superconducting temperatures.

Relative importance for each variable in the best model was calculated as the average of the selected feature importance. All models were developed using RapidMiner 9.3 [33].

## 4. Conclusions

In this paper, we analyzed a recently published dataset and related predictive models for the critical temperatures of inorganic superconductors. We have found that the initial dataset contained duplicates because the dataset contained a compilation of Tc measurements reported by different research teams and the data were not thoroughly cleaned and annotated. We suggested that collected data shall not be used in a present form along with the reported model because of the mentioned quality issues. We have profiled and cleaned the dataset and compared the efficiency of different attribute selection techniques.

Developed models allowed us to effectively predict specific critical temperatures of superconducting materials. We suggest that the models could be used to guide a data-informed search for new superconductors with a tailored value of the superconductivity temperature.

We demonstrated that the predictive quality of our models surpassed the quality of models by the author of the initial dataset. Specifically, our best model had a lower root-mean-square error and an absolute error (by 6.03% and 9.12%, respectively). We primarily focused on the optimization of XGBoost models, however, even without fine-tuning, we observed that random forest and neural networks are also promising approaches for this data set. In our future endeavors, we plan to develop a set of superconductivity models using these techniques.

## Figures and Tables

**Table 1:** Characteristics of MLR models.

Preprocessing	Attribute Selection	Performance		
		R^2^	RMSE	AE
Cleaned Dataset	n/a	0.726 ± 0.012	17.664 ± 0.279	13.317 ± 0.194
	weight by relief	0.611 ± 0.017	21.038 ± 0.490	16.286 ± 0.374
	weight by PCA	0.606 ± 0.016	21.170 ± 0.453	16.131 ± 0.326
	weight by correlation	0.618 ± 0.011	20.860 ± 0.372	16.060 ± 0.239
Correlations Removed	n/a	0.699 ± 0.009	18.505 ± 0.348	14.185 ± 0.265
	weight by relief	0.657 ± 0.021	19.771 ± 0.521	14.957 ± 0.391
	weight by PCA	0.576 ± 0.011	21.957 ± 0.243	17.339 ± 0.243
	weight by correlation	0.610 ± 0.006	21.063 ± 0.236	16.760 ± 0.165
No Outliers	**n/a** *	**0.734 ± 0.007**	**17.414 ± 0.251**	**13.124 ± 0.241**
	weigh by relief	0.607 ± 0.013	21.199 ± 0.349	16.351 ± 0.342
	weight by PCA	0.616 ± 0.012	20.936 ± 0.289	15.927 ± 0.262
	weight by correlation	0.626 ± 0.014	20.682 ± 0.347	15.882 ± 0.239
Correlations Removed, No Outliers	n/a	0.708 ± 0.016	18.244 ± 0.435	13.983 ± 0.411
	weight by relief	0.603 ± 0.017	21.310 ± 0.378	16.631 ± 0.347
	weight by PCA	0.585 ± 0.010	21.761 ± 0.367	17.163 ± 0.270
	weight by correlation	0.619 ± 0.016	20.867 ± 0.323	16.578 ± 0.293

* The best model is marked in bold.

**Table 2:** Characteristics of XGBoost models.

Preprocessing	Attribute Selection	Performance		
		R^2^	RMSE	AE
Cleaned Dataset	n/a	0.840 ± 0.011	14.376 ±0.346	10.515 ± 0.269
	weight by relief	0.801 ± 0.015	15.774 ± 0.467	11.489 ± 0.366
	weight by PCA	0.808 ± 0.007	15.576 ± 0.319	11.354 ± 0.143
	weight by correlation	0.803 ± 0.009	15.715 ± 0.315	11.442 ± 0.231
Correlations Removed	n/a	0.831 ± 0.012	14.718 ± 0.441	10.704 ± 0.309
	weight by relief	0.810 ± 0.011	15.486 ± 0.406	11.356 ± 0.193
	weight by PCA	0.799 ± 0.006	15.864 ± 0.247	11.441 ± 0.220
	weight by correlation	0.814 ± 0.006	15.337 ± 0.273	11.143 ± 0.173
No Outliers	**n/a** *	**0.847 ± 0.009**	**14.132 ± 0.347**	**10.314 ± 0.260**
	weigh by relief	0.810 ± 0.014	15.473 ± 0.344	11.250 ± 0.226
	weight by PCA	0.812 ± 0.007	15.424 ± 0.291	11.238 ± 0.191
	weight by correlation	0.810 ± 0.012	15.494 ± 0.250	11.222 ± 0.181
Correlations Removed, No Outliers	n/a	0.839 ± 0.012	14.428 ± 0.428	10.472 ± 0.301
	weight by relief	0.817 ± 0.014	15.237 ± 0.349	11.113 ± 0.245
	weight by PCA	0.803 ± 0.015	15.756 ± 0.428	11.337 ± 0.266
	weight by correlation	0.820 ± 0.016	15.114 ± 0.463	10.969 ± 0.280

* The best model is marked in bold.

**Table 3:** Characteristics of RF models.

Preprocessing	Attribute Selection	Performance		
		R^2^	RMSE	AE
Cleaned Dataset	n/a	0.863 ± 0.010	12.614 ± 0.466	8.351 ± 0.300
	weight by relief	0.836 ± 0.005	13.745 ± 0.239	9.105 ± 0.171
	weight by PCA	0.844 ± 0.007	13.410 ± 0.315	8.815 ± 0.150
	weight by correlation	0.851 ± 0.007	13.119 ± 0.194	8.643 ± 0.166
Correlations Removed	n/a	0.855 ± 0.011	12.965 ± 0.490	8.591 ± 0.315
	weight by relief	0.830 ± 0.014	13.987 ± 0.470	9.308 ± 0.249
	weight by PCA	0.837 ± 0.011	13.715 ± 0.354	9.010 ± 0.203
	weight by correlation	0.846 ± 0.009	13.331 ± 0.391	8.788 ± 0.202
No Outliers	**n/a** *	**0.868 ± 0.007**	**12.399 ± 0.247**	**8.180 ± 0.165**
	weigh by relief	0.848 ± 0.011	13.278 ± 0.439	8.748 ± 0.276
	weight by PCA	0.849 ± 0.010	13.224 ± 0.496	8.670 ± 0.313
	weight by correlation	0.856 ± 0.007	12.893 ± 0.251	8.431 ± 0.134
Correlations Removed, No Outliers	n/a	0.859 ± 0.014	12.790 ± 0.371	8.426 ± 0.177
	weight by relief	0.848 ± 0.017	13.266 ± 0.558	8.789 ± 0.277
	weight by PCA	0.843 ± 0.010	13.497 ± 0.415	8.827 ± 0.229
	weight by correlation	0.853 ± 0.015	13.063 ± 0.474	8.579 ± 0.230

* The best model is marked in bold.

**Table 4:** Characteristics of NN models.

Preprocessing	Attribute Selection	Performance		
		R^2^	RMSE	AE
Cleaned Dataset	n/a	0.837 ± 0.012	14.194 ± 0.696	9.619 ± 0.426
	weight by relief	0.746 ± 0.013	17.685 ± 0.603	12.667 ± 0.755
	weight by PCA	0.763 ± 0.012	16.902 ± 0.866	11.906 ± 1.058
	weight by correlation	0.769 ± 0.011	16.857 ± 1.009	12.028 ± 1.167
Correlations Removed	n/a	0.831 ± 0.009	14.637 ± 0.848	10.379 ± 0.999
	weight by relief	0.783 ± 0.019	16.496 ± 1.023	11.700 ± 1.117
	weight by PCA	0.766 ± 0.016	17.086 ± 0.942	12.249 ± 0.987
	weight by correlation	0.780 ± 0.012	16.746 ± 1.231	12.054 ± 1.343
No Outliers	**n/a** *	**0.842 ± 0.007**	**14.186 ± 0.794**	**10.021 ± 1.137**
	weigh by relief	0.755 ± 0.013	17.460 ± 0.773	12.497 ± 1.069
	weight by PCA	0.773 ± 0.013	16.888 ± 0.942	12.287 ± 1.019
	weight by correlation	0.774 ± 0.013	16.805 ± 0.937	12.004 ± 1.007
Correlations Removed, No Outliers	n/a	0.834 ± 0.010	13.996 ± 0.332	9.369 ± 0.305
	weight by relief	0.777 ± 0.010	16.541 ± 0.599	11.817 ± 0.726
	weight by PCA	0.775 ± 0.012	16.858 ± 1.206	12.016 ± 1.566
	weight by correlation	0.793 ± 0.012	16.394 ± 1.532	11.916 ± 2.028

* The best model is marked in bold.

**Table 5:** Characteristics of models that use aggregated attributes.

Preprocessing	Performance	Algorithm			
		MLR	XGBoost	RF	NN
Aggregation Only ^1^	R^2^	0.542 ± 0.014	0.768 ± 0.014	0.825 ± 0.008	0.688 ± 0.013
	RMSE	0.677 ± 0.012	0.501 ± 0.013	0.421 ± 0.012	0.566 ± 0.018
	AE	0.535 ± 0.008	0.364 ± 0.011	0.278 ± 0.007	0.408 ± 0.023
Aggregation Only ^1^, No outliers	R^2^	0.530 ± 0.013	0.780 ± 0.012	0.834 ± 0.012	0.691 ± 0.021
	RMSE	0.673 ± 0.017	0.492 ± 0.012	0.412 ± 0.015	0.574 ± 0.023
	AE	0.551 ± 0.016	0.356 ± 0.009	0.270 ± 0.010	0.419 ± 0.024
Aggregation, Merged Attributes	R^2^	0.726 ± 0.011	0.840 ± 0.012	0.863 ± 0.011	0.836 ± 0.009
	RMSE	17.657 ± 0.421	14.376 ± 0.433	12.615 ± 0.433	14.224 ± 0.591
	AE	13.312 ± 0.263	10.490 ± 0.293	8.339 ± 0.261	9.932 ± 0.836
Aggregation, Merged Attributes, No outliers	R^2^	0.735 ± 0.006	0.846 ± 0.012	0.867 ± 0.012	0.844 ± 0.011
	RMSE	17.409 ± 0.300	14.126 ± 0.378	12.405 ± 0.524	13.624 ± 0.391
	AE	13.121 ± 0.293	10.279 ± 0.318	8.186 ± 0.377	9.469 ± 0.504

^1^ These models are based on normalized attributes.

**Table 6:** Characteristics of optimized XGBoost models.

Preprocessing	Attribute selection	Performance		
		R^2^	RMSE	AE
Original Dataset (with Duplicates)	n/a (XGBoost model from [1])	0.92	9.5	-
	n/a	0.926 ± 0.004	9.344 ± 0.289	5.142 ± 0.147
	weight by relief	0.922 ± 0.005	9.544 ± 0.372	5.313 ± 0.160
	weight by PCA	0.922 ± 0.007	9.551 ± 0.357	5.346 ± 0.107
	weight by correlation	0.923 ± 0.007	9.494 ± 0.504	5.297 ± 0.168
Cleaned Dataset	n/a	0.923 ± 0.005	9.365 ± 0.329	5.168 ± 0.110
	weight by relief	0.914 ± 0.009	9.882 ± 0.518	5.504 ± 0.221
	weight by PCA	0.917 ± 0.009	9.737 ± 0.476	5.513 ± 0.248
	weight by correlation	0.917 ± 0.009	9.683 ± 0.492	5.510 ± 0.141
Correlations Removed	n/a	0.925 ± 0.005	9.265 ± 0.244	5.170 ± 0.190
	weight by relief	0.920 ± 0.009	9.557 ± 0.511	5.377 ± 0.256
	weight by PCA	0.918 ± 0.008	9.665 ± 0.442	5.463 ± 0.189
	weight by correlation	0.919 ± 0.009	9.613 ± 0.544	5.424 ± 0.235
No Outliers	**n/a** *	**0.930 ± 0.012**	**8.927 ± 0.689**	**4.975 ± 0.259**
	weight by relief	0.921 ± 0.007	9.497 ± 0.417	5.334 ± 0.169
	weight by PCA	0.920 ± 0.007	9.557 ± 0.388	5.408 ± 0.211
	weight by correlation	0.922 ± 0.010	9.444 ± 0.593	5.354 ± 0.285
Correlations Removed, No Outliers	n/a	0.929 ± 0.005	9.012 ± 0.319	5.030 ± 0.121
	weight by relief	0.924 ± 0.004	9.336 ± 0.242	5.296 ± 0.121
	weight by PCA	0.922 ± 0.006	9.413 ± 0.379	5.332 ± 0.196
	weight by correlation	0.921 ± 0.011	9.477 ± 0.659	5.334 ± 0.279

* The best model is marked in bold.

**Table 7:** Importance of attributes in the best XGBoost model.

Attribute ^1^	Relative Importance	Scaled Importance
range_ThermalConductivity	47,722,904.0	1.000
wtd_gmean_ThermalConductivity	10,336,861.0	0.217
range_atomic_radius	3,051,781.3	0.064
range_atomic_mass	2,503,977.0	0.052
range_fie	2,469,144.3	0.052
wtd_range_fie	1,768,628.4	0.037
wtd_mean_atomic_mass	1,551,901.4	0.033
mean_Density	1,533,498.8	0.032
gmean_atomic_radius	1,522,213.5	0.032
wtd_range_atomic_radius	1,455,983.8	0.031
wtd_mean_Density	890,073.5	0.019
wtd_std_fie	832,274.6	0.017
wtd_mean_atomic_radius	832,100.6	0.017
mean_fie	792,180.4	0.017
range_Density	744,477.6	0.016
range_ElectronAffinity	720,590.1	0.015
gmean_ThermalConductivity	670,280.3	0.014
mean_atomic_mass	664,245.5	0.014
gmean_atomic_mass	412,535.3	0.009
wtd_gmean_Density	344,775.4	0.007

^1^ in names of attributes: wtd = weighted, gmean = geometric mean, std = standard deviation, fie = first ionization energy.

## Supplementary Materials

The following are available online, Table S1: Description of the initial data set, Table S2: Description of the cleaned data set, Figure S2: Cumulative variance of added variables in PCA modeling, Model S4: RapidMiner archive.

## Sample Availability

Samples of the compounds are not available from the authors.

## Abbreviations

AE	absolute error
MLR	multiple linear regression
NN	neural network
PCA	principal component analysis
RF	random forests
PLS	projections to latent structures
RMSE	root mean squared error
XGBoost	gradient boosted decision trees
