# Supplementary material for: Predictive Modeling of Critical Temperatures in Superconducting Materials
Source: Molecules. 2020 Dec 22;26(1):8. doi: 10.3390/molecules26010008 (PMC7792800; doi:10.3390/molecules26010008)
Supplement: Supplementary file 1 [file molecules-26-00008-s001.pdf]

molecules

MDPI

Supplementary materials

Predictive Modeling of Critical Temperatures in Superconducting Materials

Natalia Sizochenko [1,2,3] * and Markus Hofmann [1]

1 Department of Informatics, Blanchardstown Campus, Technological University Dublin, Dublin 15, Ireland; markus.hofmann@tudublin.ie
2 Department of Informatics, Postdoctoral Institute for Computational Studies, Enfield, NH 03748, USA; natalia.sizochenko@picomps.org
3 Previous address: Department of Computer Science, Dartmouth College, Hanover, NH 03755, USA
* Correspondence: natalia.sizochenko@picomps.org

Table S1. Description of the initial data set

Attribute name	Description	Mean	Min	Max	Standard deviation
number_of_elements	number of unique elements	4.12	1.00	9.00	1.44
mean_atomic_mass	attributes that represent atomic mass	87.56	6.94	208.98	29.68
wtd_mean_atomic_mass		72.99	6.42	208.98	33.49
gmean_atomic_mass		71.29	5.32	208.98	31.03
wtd_gmean_atomic_mass		58.54	1.96	208.98	36.65
range_atomic_mass		115.60	0.00	207.97	54.63
wtd_range_atomic_mass		33.23	0.00	205.59	26.97
std_atomic_mass		44.39	0.00	101.02	20.03
wtd_std_atomic_mass		41.45	0.00	101.02	19.98
entropy_atomic_mass		1.17	0.00	1.98	0.36
wtd_entropy_atomic_mass		1.06	0.00	1.96	0.40
mean_fie	attributes that represent first ionization energy	769.61	375.50	1,313.10	87.49
wtd_mean_fie		870.44	375.50	1,348.03	143.27
gmean_fie		737.47	375.50	1,313.10	78.33
wtd_gmean_fie		832.77	375.50	1,327.59	119.77
entropy_fie		1.30	0.00	2.16	0.38
wtd_entropy_fie		0.93	0.00	2.04	0.33
range_fie		572.22	0.00	1,304.50	309.61
wtd_range_fie		483.52	0.00	1,251.86	224.04
std_fie		215.63	0.00	499.67	109.96
wtd_std_fie		224.05	0.00	479.16	127.92
mean_atomic_radius	attributes that represent atomic radius	157.98	48.00	298.00	20.15
wtd_mean_atomic_radius		134.72	48.00	298.00	28.80
gmean_atomic_radius		144.45	48.00	298.00	22.09

wtd_gmean_atomic_radius		120.99	48.00	298.00	35.84
entropy_atomic_radius		1.27	0.00	2.14	0.38
wtd_entropy_atomic_radius		1.13	0.00	1.90	0.41
range_atomic_radius		139.33	0.00	256.00	67.27
wtd_range_atomic_radius		51.37	0.00	240.16	35.02
std_atomic_radius		51.60	0.00	115.50	22.90
wtd_std_atomic_radius		52.34	0.00	97.14	25.29
mean_Density	attributes that represent density	6,111.47	1.43	22,590.00	2,846.72
wtd_mean_Density		5,267.19	1.43	22,590.00	3,221.24
gmean_Density		3,460.69	1.43	22,590.00	3,703.17
wtd_gmean_Density		3,117.24	0.69	22,590.00	3,975.03
entropy_Density		1.07	0.00	1.95	0.34
wtd_entropy_Density		0.86	0.00	1.70	0.32
range_Density		8,665.44	0.00	2,2588.57	4097.03
wtd_range_Density		2,902.74	0.00	2,2434.16	2398.41
std_Density		3,416.91	0.00	10,724.37	1,673.59
wtd_std_Density		3,319.17	0.00	10,410.93	1,611.76
mean_ElectronAffinity	attributes that represent electron affinity	76.88	1.50	326.10	27.70
wtd_mean_ElectronAffinity		92.72	1.50	326.10	32.28
gmean_ElectronAffinity		54.36	1.50	326.10	29.01
wtd_gmean_ElectronAffinity		72.42	1.50	326.10	31.65
entropy_ElectronAffinity		1.07	0.00	1.77	0.34
wtd_entropy_ElectronAffinity		0.77	0.00	1.68	0.29
range_ElectronAffinity		120.73	0.00	349.00	58.70
wtd_range_ElectronAffinity		59.33	0.00	218.70	28.62
std_ElectronAffinity		48.91	0.00	162.90	21.74
wtd_std_ElectronAffinity		44.41	0.00	169.08	20.43
mean_FusionHeat	attributes that represent fusion heat	14.30	0.22	105.00	11.30
wtd_mean_FusionHeat		13.85	0.22	105.00	14.28
gmean_FusionHeat		10.14	0.22	105.00	10.07
wtd_gmean_FusionHeat		10.14	0.22	105.00	13.13
entropy_FusionHeat		1.09	0.00	2.03	0.38
wtd_entropy_FusionHeat		0.91	0.00	1.75	0.37
range_FusionHeat		21.14	0.00	104.78	20.37
wtd_range_FusionHeat		8.22	0.00	102.68	11.41
std_FusionHeat		8.32	0.00	51.64	8.67
wtd_std_FusionHeat		7.72	0.00	51.68	7.29
mean_ThermalConductivity	attributes that represent thermal conductivity	89.71	0.03	332.50	38.52
wtd_mean_ThermalConductivity		81.55	0.03	406.96	45.52
gmean_ThermalConductivity		29.84	0.03	317.88	34.06
wtd_gmean_ThermalConductivity		27.31	0.02	376.03	40.19
entropy_ThermalConductivity		0.73	0.00	1.63	0.33
wtd_entropy_ThermalConductivity		0.54	0.00	1.61	0.32
range_ThermalConductivity		250.89	0.00	429.97	158.70

wtd_range_ThermalConductivity		62.03	0.00	401.44	43.12
std_ThermalConductivity		98.94	0.00	214.99	60.14
wtd_std_ThermalConductivity		96.23	0.00	213.30	63.71
mean_Valence	attributes that represent valence	3.20	1.00	7.00	1.04
wtd_mean_Valence		3.15	1.00	7.00	1.19
gmean_Valence		3.06	1.00	7.00	1.05
wtd_gmean_Valence		3.06	1.00	7.00	1.17
entropy_Valence		1.30	0.00	2.14	0.39
wtd_entropy_Valence		1.05	0.00	1.95	0.38
range_Valence		2.04	0.00	6.00	1.24
wtd_range_Valence		1.48	0.00	6.99	0.98
std_Valence		0.84	0.00	3.00	0.48
wtd_std_Valence		0.67	0.00	3.00	0.46
critical_temp	critical temperature T_c	34.42	0.00	185.00	34.25

Table S2. Description of the cleaned data set

Attribute name	Description	Mean	Min	Max	Standard deviation
number_of_elements	number of unique elements	4.24	1.00	9.00	1.47
mean_atomic_mass	attributes that represent atomic mass	88.87	6.94	208.98	29.69
wtd_mean_atomic_mass		74.29	6.42	208.98	34.01
gmean_atomic_mass		72.59	5.32	208.98	31.25
wtd_gmean_atomic_mass		59.99	1.96	208.98	37.35
entropy_atomic_mass		1.20	0.00	1.98	0.35
wtd_entropy_atomic_mass		1.08	0.00	1.96	0.41
range_atomic_mass		116.90	0.00	207.97	53.93
wtd_range_atomic_mass		35.13	0.00	205.59	28.63
std_atomic_mass		44.80	0.00	101.02	19.67
wtd_std_atomic_mass		41.36	0.00	101.02	19.73
mean_fie	attributes that represent first ionization energy	765.68	375.50	1,313.10	86.85
wtd_mean_fie		864.99	375.50	1,348.03	143.88
gmean_fie		734.70	375.50	1,313.10	77.63
wtd_gmean_fie		828.26	375.50	1,327.59	120.21
entropy_fie		1.33	0.00	2.16	0.37
wtd_entropy_fie		0.94	0.00	2.04	0.33
range_fie		566.02	0.00	1,304.50	310.11
wtd_range_fie		488.12	0.00	1,251.86	216.49
std_fie		211.34	0.00	499.67	108.89
wtd_std_fie		220.37	0.00	479.16	128.20
mean_atomic_radius	attributes that represent atomic radius	158.87	48.00	298.00	19.79
wtd_mean_atomic_radius		136.18	48.00	298.00	28.94
gmean_atomic_radius		145.83	48.00	298.00	21.90

wtd_gmean_atomic_radius		122.75	48.00	298.00	36.18
entropy_atomic_radius		1.30	0.00	2.14	0.36
wtd_entropy_atomic_radius		1.14	0.00	1.90	0.41
range_atomic_radius		137.93	0.00	256.00	67.36
wtd_range_atomic_radius		54.81	0.00	240.16	36.70
std_atomic_radius		50.59	0.00	115.50	22.56
wtd_std_atomic_radius		51.57	0.00	97.14	25.34
mean_Density	attributes that represent density	6,259.56	1.43	22,590.00	2,914.58
wtd_mean_Density		5,415.38	1.43	22,590.00	3,329.27
gmean_Density		3,629.55	1.43	22,590.00	3,787.90
wtd_gmean_Density		3,278.54	0.69	22,590.00	4,099.13
entropy_Density		1.11	0.00	1.95	0.33
wtd_entropy_Density		0.87	0.00	1.70	0.32
range_Density		8,825.62	0.00	22,588.57	4,081.38
wtd_range_Density		3,074.60	0.00	22,434.16	2,567.74
std_Density		3,466.03	0.00	10,724.37	1,658.21
wtd_std_Density		3,339.55	0.00	10,410.93	1,598.58
mean_ElectronAffinity	attributes that represent electron affinity	76.25	1.50	326.10	27.67
wtd_mean_ElectronAffinity		92.27	1.50	326.10	32.37
gmean_ElectronAffinity		53.59	1.50	326.10	28.83
wtd_gmean_ElectronAffinity		71.71	1.50	326.10	31.55
entropy_ElectronAffinity		1.10	0.00	1.77	0.33
wtd_entropy_ElectronAffinity		0.78	0.00	1.68	0.28
range_ElectronAffinity		122.12	0.00	349.00	59.16
wtd_range_ElectronAffinity		59.71	0.00	218.70	28.35
std_ElectronAffinity		49.09	0.00	162.90	21.81
wtd_std_ElectronAffinity		44.68	0.00	169.08	20.87
mean_FusionHeat	attributes that represent fusion heat	14.19	0.22	105.00	10.54
wtd_mean_FusionHeat		13.77	0.22	105.00	13.48
gmean_FusionHeat		10.17	0.22	105.00	9.49
wtd_gmean_FusionHeat		10.13	0.22	105.00	12.41
entropy_FusionHeat		1.13	0.00	2.03	0.36
wtd_entropy_FusionHeat		0.93	0.00	1.75	0.37
range_FusionHeat		21.15	0.00	104.78	20.06
wtd_range_FusionHeat		8.34	0.00	102.68	10.84
std_FusionHeat		8.23	0.00	51.64	8.37
wtd_std_FusionHeat		7.65	0.00	51.68	7.24
mean_ThermalConductivity	attributes that represent thermal conductivity	89.47	0.03	332.50	38.61
wtd_mean_ThermalConductivity		82.39	0.03	406.96	46.53
gmean_ThermalConductivity		30.47	0.03	317.88	33.94
wtd_gmean_ThermalConductivity		28.07	0.02	376.03	41.02
entropy_ThermalConductivity		0.77	0.00	1.63	0.31
wtd_entropy_ThermalConductivity		0.56	0.00	1.61	0.32
range_ThermalConductivity		251.07	0.00	429.97	157.29

wtd_range_ThermalConductivity		63.04	0.00	401.44	43.83
std_ThermalConductivity		97.98	0.00	214.99	58.62
wtd_std_ThermalConductivity		95.98	0.00	213.30	63.45
mean_Valence	attributes that represent valence	3.24	1.00	7.00	1.05
wtd_mean_Valence		3.19	1.00	7.00	1.21
gmean_Valence		3.10	1.00	7.00	1.05
wtd_gmean_Valence		3.09	1.00	7.00	1.19
entropy_Valence		1.33	0.00	2.14	0.38
wtd_entropy_Valence		1.07	0.00	1.95	0.38
range_Valence		2.10	0.00	6.00	1.23
wtd_range_Valence		1.56	0.00	6.99	1.02
std_Valence		0.86	0.00	3.00	0.48
wtd_std_Valence		0.68	0.00	3.00	0.45
critical_temp	critical temperature T_c	33.31	0.00	143.00	33.74

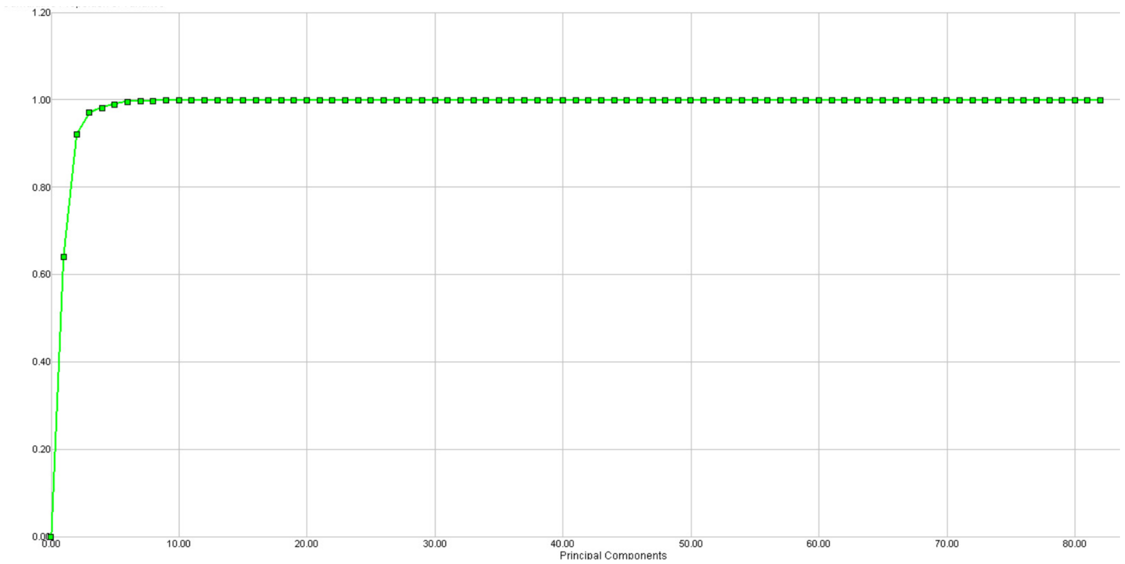

Figure S3. Cumulative variance of added variables in PCA modeling

Model S4. RapidMiner archive: https://drive.google.com/drive/folders/1CfAy_XTCgOf1un-lAEBsXLduKJErtxH8
